# MitoQ Protects Against Oxidative Stress-Induced Mitochondrial Dysregulation in Human Cardiomyocytes

**DOI:** 10.1016/j.jmccpl.2025.100469

**Published:** 2025-06-26

**Authors:** Alex M. Parker, Jarmon G. Lees, Mitchel Tate, Ren J. Phang, Anida Velagic, Minh Deo, Tayla Bishop, Thomas Krieg, Michael P. Murphy, Shiang Y. Lim, Miles J. De Blasio, Rebecca H. Ritchie

**Affiliations:** a Monash Institute of Pharmaceutical Sciences, Monash University, Melbourne, VIC, Australia; b O'Brien Institute Department, St Vincent's Institute of Medical Research, Melbourne, VIC, Australia; c Department of Medicine, University of Cambridge, Cambridge Biomedical Campus, United Kingdom; d MRC Mitochondrial Biology Unit, University of Cambridge, Cambridge Biomedical Campus, United Kingdom; e Department of Medicine and Surgery, University of Melbourne, Melbourne, VIC, Australia; f National Heart Research Institute Singapore, National Heart Centre, 5 Hospital Drive, 169609, Singapore; g Baker Heart and Diabetes Institute, Melbourne, VIC, Australia

**Keywords:** Mitoquinone mesylate, Dodecyl-triphenylphosphonium, Mitochondrial function, Reactive oxygen species, Induced pluripotent stem cells, Cardiomyocytes

## Abstract

The overproduction of reactive oxygen species (ROS) and mitochondrial dysregulation are regarded as key mechanisms in the progression of cardiac remodelling in cardiometabolic diseases including heart failure. Conventional treatments are often ineffective as they do not specifically target the underlying pathological mechanisms. Mitoquinone mesylate (MitoQ), a mitochondrial-targeted antioxidant has been reported to be protective against vascular dysfunction in hypertension, diabetic kidney disease and alcohol-induced liver damage. However, the cardioprotective potential of MitoQ to limit oxidative stress-induced mitochondrial remodelling in cardiomyocytes has not been fully resolved. We sought to investigate the effect of MitoQ and its mitochondrial-targeting moiety dodecyl-triphenylphosphonium (dTPP) on hydrogen peroxide-induced overproduction of ROS, mitochondrial dysregulation and cell death in H9C2 rat cardiomyoblasts (H9C2-rCM) and human induced pluripotent stem cell-derived cardiomyocytes (hiPSC-CM). Cardiomyocytes were exposed to acute or chronic treatment (5–60 min or 48 h) of vehicle control (0.0001 % Ultrapure Milli-Q water), hydrogen peroxide (100 μM) ± MitoQ (1 μM) or dTPP (1 μM) control. Hydrogen peroxide-induced overproduction of ROS, extracellular superoxide, mitochondrial ROS, mitochondrial hyperpolarisation and cell death were significantly blunted by MitoQ, but not dTPP, suggesting that the coenzyme Q_10_ moiety of MitoQ is protective under these conditions. Interestingly, both MitoQ and dTPP exhibited a pro-mitochondrial fusion effect by preserving mitochondrial network and reducing mitochondrial fragmentation in oxidative stress conditions. Overall, our findings confirm the cytoprotective potential of MitoQ to limit oxidative stress-induced adverse mitochondrial remodelling and dysregulation that is clinically observed in cardiometabolic-induced cardiac dysfunction in the failing heart.

## Highlights

• Oxidative stress is a key driver of mitochondrial dysregulation and cell death in cardiomyocytes.
• MitoQ exhibits cytoprotection against elevated ROS production in human cardiomyocytes.
• Mitochondrial structure and regulation were preserved in human cardiomyocytes with MitoQ treatment.

## Graphical abstract

BioRender Scientific Image and Illustration Software were used to generate the graphical abstract.

**Figure:**
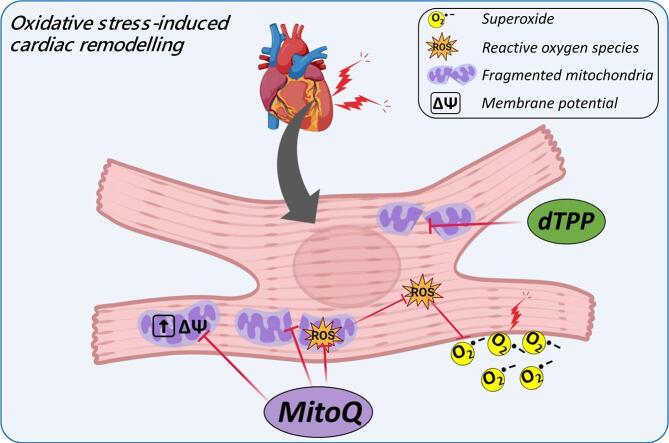

## 1 Introduction

Heart failure is regarded as a global pandemic affecting ∼64 million adults worldwide, and this is projected to rise by 46 % by 2030 [1]. This is accompanied by a heavy economic burden with a global annual expenditure of ∼USD$108 billion [2]. The aetiology of heart failure is complex and multifactorial in nature [3]. Several comorbidities have been associated with heart failure including, myocardial infarction, ischemic heart disease, dilated cardiomyopathy, hypertension, diabetes, obesity and atrial fibrillation [4,5]. Patients with heart failure exhibit impaired ventricular filling and/or reduced cardiac output, caused by cardiac remodelling including hypertrophy, fibrosis, inflammation, altered cardiomyocyte contraction and relaxation [1,6]. Despite the scientific progress in therapies over the past 30 years, patients with heart failure still face a poor prognosis with 1-year mortality ranging from 15 to 30 %, and 5-year mortality of up to 75 % [7]. Conventional treatments are ineffective, partially because they are not specifically tailored to target the underlying pathological mechanisms of heart failure [8].

Reactive oxygen species (ROS)-induced oxidative stress and mitochondrial dysregulation have been well-established as key mechanisms that drive the progression of heart failure [6]. Myocardial mitochondria are a substantial source of ROS, accounting for ∼90 % of cellular ROS in the failing heart [9,10]. Dysregulation of mitochondrial substrate utilisation in heart failure can cause uncoupled singlet electrons to leak from the mitochondrial respiratory chain, causing the reduction of oxygen molecules to form mitochondrial ROS, leading to oxidative stress [11,12]. This has been reported in myocardial biopsies of patients with heart failure exhibiting excess mitochondrial ROS such as hydrogen peroxide [13]. These conditions can damage components of the mitochondria and further potentiate electron leakage, ROS production and oxidative damage to vital cellular components in the myocardium [10,14]. This is evidenced in the right ventricular sections of patients with heart failure exhibiting increased oxidative DNA modifications *via* immunoreactivity for 8-OHdG, indicative of oxidative damage [15]. Targeting the overproduction of ROS has become a promising approach in the treatment of heart failure due to a plethora of studies reporting the causal interplay between oxidative stress and mitochondrial dysregulation [[16], [17], [18]]. Antioxidants such as coenzyme Q_10_ have shown therapeutic potential to combat oxidative stress in heart failure [19]. However, due to its large molecular weight and hydrophobicity, coenzyme Q_10_ has poor bioavailability and is unable to cross the mitochondrial membrane, which limits its therapeutic potential [20,21]. These limitations were addressed with the development of the mitochondrial-targeting coenzyme Q_10_, mitoquinone mesylate (MitoQ) [22]. MitoQ was synthesised by conjugating the coenzyme Q_10_ antioxidant moiety to dodecyl-triphenylphosphonium (dTPP), a positively charged lipophilic cation moiety, a combination designed to therapeutically target mitochondrial oxidative damage [23,24]. The dTPP cation circumvents the hydrophobic nature of coenzyme Q_10_ and allows molecular uptake into the mitochondria up to ∼1000-fold compared to the cytoplasm, resulting in more efficient delivery of coenzyme Q_10_ into the mitochondria [23,25,26]. MitoQ has since been investigated *in vitro* and *in vivo* in a broad spectrum of diseases including cardiovascular disease, diabetic kidney disease, diabetic cardiomyopathy and obesity [[27], [28], [29], [30]]. However, the protective role of MitoQ in limiting oxidative stress-induced injury in the failing heart has not been fully resolved. Notably, a large proportion of studies were not performed with dTPP control, a molecule with similar hydrophobicity to MitoQ but without the coenzyme Q_10_ antioxidant moiety. Therefore, the contribution of dTPP to the therapeutic effect of MitoQ is still unclear [27,[29], [30], [31], [32], [33]].

In the present study, we sought to specifically evaluate the cytoprotective effects of the mitochondria-targeted antioxidant MitoQ (as distinct from its dTPP moiety) in H9C2 rat cardiomyoblasts (H9C2-rCM) and human induced pluripotent stem cell-derived cardiomyocytes (hiPSC-CM) exposed to hydrogen peroxide-induced oxidative stress in acute (5-minute exposure) and chronic (48-hour exposure) conditions.

## 2 Methods and materials

### 2.1 H9C2-rCM culture

H9C2 cardiomyoblasts (commercially-available cell line originally derived from ventricular tissues of embryonic BD1X rat hearts; H9C2-rCM; [34]) were generously provided by the Cardiac Hypertrophy Laboratory, Baker Heart and Diabetes Institute (Melbourne, Victoria). Cells were cultured in H9C2 maintenance media consisting of Dulbecco's Modified Eagle Medium (DMEM; 25 mM glucose, Thermo Fisher Scientific) supplemented with 10 % fetal bovine serum (FBS, Thermo Fisher Scientific) and 1 % penicillin/streptomycin (Thermo Fisher Scientific). The media was refreshed every 2 days. Cells were then utilised for various analyses under oxidative stress conditions (exposure to hydrogen peroxide 100 μM; Cat# 605852, Gold Cross) including extracellular superoxide, intracellular ROS and mitochondrial ROS (measured at the 5 min or 48 hr timepoints; acute and chronic time points, respectively). A complete list of cell culturing reagents is presented in Supplementary Table 1.

### 2.2 hiPSC-CM differentiation and culture

Cardiomyocytes were differentiated from human iPSCs (iPS-Foreskin-2 cell line) using a previously described protocol [35,36]. Human iPSCs were seeded onto 24 well plates and cultured in TeSR-E8 medium (Stem Cell Technologies) containing Y-27632 dihydrochloride (10 μM, Tocris Bioscience, Day -2). The medium was then replaced with TeSR-E8 medium after 24 h (Day -1). Cells were then incubated with RPMI 1640 basal medium (11 mM glucose, Thermo Fisher Scientific) containing insulin-free B27 supplement (Cat# A1895601, Thermo Fisher), CHIR99021 (10 μM, Cat# 13122 Cayman Chemical) and growth factor reduced-Matrigel (1:60 dilution, Cat# CLS356231, Sigma Aldrich, Day 0). On Day 1, cells were cultured in RPMI 1640 basal medium containing B27 supplement minus insulin. On the following day, cells were cultured in RPMI 1640 basal medium containing B27 supplement minus insulin and IWP2 (5 μM, Cat# 3533 Tocris Bioscience) for 72 h. For the following 7 days (Days 5–12), cells were maintained and medium changed every 48 h with RPMI 1640 basal medium containing B27 supplement and l-ascorbic acid 2-phosphate sesquimagnesium salt hydrate (200 μg/mL, Cat# A92902, Sigma Aldrich), referred to as cardiomyocyte media. On Day 12, cells from 1 × 24-well plate were split into 3 × 10 cm dishes (Cat# 3160, Corning) containing DMEM-F12 GlutaMax medium (Cat# 10565042, Sigma-Aldrich) supplemented with 20 % fetal calf serum (Cat# 12006C, Sigma Aldrich), MEM non-essential amino acids (100 μM, Cat# M7145 Sigma Aldrich), 2-mercaptoethanol (0.1 mM, Cat# 21985023 Thermo Fisher), penicillin/streptomycin (50 U/mL) and Y-27632 dihydrochloride (10 μM), referred to as complete medium. On day 13, the medium was replaced with RPMI medium containing B27 supplement CHIR99021 and l-ascorbic acid 2-phosphate sesquimagnesium salt hydrate (200 μg/mL). The cardiomyocyte enrichment process then began on Day 14, where cells were cultured in a glucose-free DMEM medium containing sodium L-lactate (4 mM, Cat# 867-56-1, Sigma Aldrich) until Day 19 (medium changed on Days 15 and 16). Enriched cardiomyocytes were then replaced in a complete medium for experiments. The following day (Day 20), the medium was replaced with cardiomyocyte media. Cells were then sampled and stained with cardiac troponin T (cTnT; specific marker of cardiomyocytes) and DAPI (4′,6-diamidino-2-phenylindole; fluorescent probe that binds to DNA) to assess the percentage of cardiomyocyte purity. ImageJ cell counter software was used to count the number of cTnT+ cells and cTnT- cells. Only cells that exhibited cTnT+ of 95 % or above were used for experiments (Supplementary Fig. 1). Cardiomyocytes were then utilised for various analyses under acute oxidative stress conditions (exposure to hydrogen peroxide 100 μM) including extracellular superoxide (measured at timepoint 5 min), intracellular ROS (measured at timepoint 5 min), cell death (measured at timepoint 60 min). Various measurements were also collected under chronic oxidative stress conditions (exposure to hydrogen peroxide 100 μM for 48 h) including mitochondrial ROS, mitochondrial membrane potential, mitochondrial morphology and cell death.

### 2.3 Quantification of extracellular superoxide

Extracellular superoxide levels (significantly derived from nicotinamide adenine dinucleotide phosphate [NADPH] oxidase) were assessed with a previously described luminol-based-chemiluminescent-8-amino-5-chloro-7-phenyl-pyrido[3,4d]-pyridazine1,4[2H,3H]-dione (L-012; Cat# SML2236, Sigma Aldrich) assay [37]. Cardiomyocytes (H9C2-rCM or hiPSC-CM) were seeded at 10,000 cells/well in white 96-well clear-bottom plates containing either H9C2-rCM maintenance media (for H9C2-rCM) or iPSC cardiomyocyte media (for hiPSC-CM), and maintained at 37 °C in a humidified 5 % CO_2_ incubator for 24 h. Cells were then exposed to vehicle control (0.0001 % Ultrapure Milli-Q water), MitoQ (kindly provided by Antipodean Pharmaceuticals) or dTPP (Cat# K4002, Sigma Aldrich) (both 1 μM) ± hydrogen peroxide (100 μM) for 5 min (acute oxidative stress) or 48 h (chronic oxidative stress) before being washed twice with Krebs buffer (Cat# K4002, Sigma Aldrich) and the addition of Krebs buffer containing L-012 (20 μM). Microplate was immediately placed in an Enspire microplate reader (Perkin Elmer) for chemiluminescent detection.

### 2.4 Assessment of intracellular oxidative stress

Intracellular oxidative stress was measured using 2′,7′–dichlorofluorescin diacetate (DCFDA, Cat# D399, Thermo Fisher) fluorescence [38]. Cardiomyocytes (H9C2-rCM or hiPSC-CM) were seeded at 10,000 cells/well in black 96-well clear-bottom plates containing either H9C2 maintenance media (for H9C2-rCM) or iPSC cardiomyocyte media (for hiPSC-CM), and maintained at 37 °C in a humidified 5 % CO_2_ incubator for 24 h. Cells were then exposed to vehicle control (0.0001 % Ultrapure Milli-Q water), MitoQ or dTPP (both 1 μM) ± hydrogen peroxide (100 μM) for 5 min (acute oxidative stress) or 48 h (chronic oxidative stress). Media was then replaced with phosphate-buffered saline (PBS; Cat# P4417, Sigma Aldrich) containing DCFDA (10 μM) for a 40-minute incubation at 37 °C in a humidified 5 % CO_2_ in the dark before being washed twice with PBS. PBS was then added back into the wells before fluorescence detection (485 nm/535 nm, excitation/emission) using an Enspire microplate reader (Perkin Elmer).

### 2.5 Measurement of mitochondrial ROS

Mitochondria ROS production was assessed using a commercially available fluorogenic agent, MitoSOX red (MitoSOX; Cat# M36008, Thermo Fisher) [39]. hiPSC-CM or H9C2-rCM were seeded at 20,000 cells/well in clear 48-well plates containing iPSC cardiomyocyte media and or H9C2-rCM maintenance media and maintained at 37 °C in a humidified 5 % CO_2_ incubator for 24 h. Cells were then exposed to vehicle control (0.0001 % Ultrapure Milli-Q water) or hydrogen peroxide (100 μM; chronic oxidative stress) ± MitoQ or dTPP (1 μM) for 48 h. Media was then replaced with Hanks' Balanced Salt Solution (HBSS; Cat# 14025092 Thermo Fisher) containing 1 μM MitoSOX and incubated in the dark for 20 min in a 37 °C humidified 5 % CO_2_ incubator. Both bright field and fluorescent images (excitation/emission; 510/580 nm) from 3 fields of view were immediately captured using an Olympus IX71 (Tokyo, Japan) at 200× magnification. Images were numbered at the time of acquisition to blind the investigator to experimental groups, and a total of ∼100 cells were randomly selected by the investigator for every biological replicate.

Images were later manually analysed using the method:

Corrected total cell fluorescence (CTCF = integrated density − (Cellular area × mean fluorescence of background) method using ImageJ software.

### 2.6 Assessment of mitochondrial membrane potential

Mitochondria membrane potential was measured with a commercial Tetramethylrhodamine Methyl Ester (TMRM) assay kit (Cat# T668, Thermo Fisher) [40]. hiPSC-CM were seeded at 20,000 cells/well in clear 48-well plates containing iPSC cardiomyocyte media, and maintained at 37 °C in a humidified 5 % CO_2_ incubator for 24 h. hiPSC-CM were then exposed to vehicle control (0.0001 % Ultrapure Milli-Q water) or hydrogen peroxide (100 μM) ± MitoQ or dTPP (1 μM) for 48 h. Cells were incubated with DMEM media containing TMRM (1 nM; final concentration) in the dark for 20 min in a 37 °C humidified 5 % CO_2_ incubator. Both bright field and fluorescent images (wavelength, 552/574 nm) from 3 fields of view were immediately captured using an Olympus IX71 (Tokyo, Japan) at 200× magnification. Images were numbered at the time of acquisition to blind the investigator to experimental groups, and a total of ∼100 cells were randomly selected by the investigator for every biological replicate.

Images were later manually analysed using the method:

Corrected total cell fluorescence (CTCF = integrated density − (Cellular area × mean fluorescence of background) method using ImageJ software.

### 2.7 Mitochondrial function analysis

Assessment of mitochondrial function was carried out to assess the respiratory chain efficiency between electron flow and adenosine triphosphate (ATP) synthesis *via* ATP synthase (ATP-linked respiration) previously described [41]. H9C2-rCM was seeded at 10,000 cells/well in 96 Seahorse XF96 tissue culture Microplates (Seahorse Bioscience, North Billerica, MA, USA). containing H9C2-rCM maintenance media and maintained at 37 °C in a humidified 5 % CO_2_ incubator for 24 h. H9C2-rCM were then exposed to vehicle control (0.0001 % Ultrapure Milli-Q water) or hydrogen peroxide (100 μM; chronic oxidative stress) ± MitoQ or dTPP (1 μM) for 48 h. Cells were washed with mitochondrial assay solution (MAS; mannitol 660 mM, sucrose 210 mM, potassium phosphate 30 mM, magnesium chloride 15 mM, HEPES buffer [4-(2-Hydroxyethyl)-1-piperazineethanesulfonic acid] 6 mM, EGTA [Ethylene glycol-bis (β-aminoethyl ether)-N,N,N′,N′-tetra acetic acid] 3 mM, fatty acid free bovine serum albumin 0.6 % (*w*/*v*)). Media was then replaced with MAS enriched with sodium pyruvate 10 mM, 1 mM malate and succinate 10 mM and inserted into Seahorse (XF) 96 Analyzer (Seahorse Bioscience, North Billerica, MA, USA). Adenosine diphosphate 40 mM, oligomycin 20 μM, Trifluoromethoxy carbonyl cyanide phenylhydrazone [FCCP] 40 μM and antimycin A 25 μM were then injected sequentially, with oxygen consumption rate (OCR) measurements recorded after each injection. OCR values were normalised to the number of cells per well, captured using Operetta® CLS™ high-content analysis system (PerkinElmer), cell segmentation digital phase contrast mode.

### 2.8 Assessment of mitochondrial morphology

Mitochondrial morphology was assessed using Hsp60 immunostaining (Cat# ab46798, Abcam) on cells cultured on coverslips [42]. After exposure to vehicle control (0.0001 % Ultrapure Milli-Q water) or hydrogen peroxide (100 μM) ± MitoQ or dTPP (1 μM) for 48 h, hiPSC-CM were fixed (4 % paraformaldehyde; Sigma-Aldrich) and permeabilised (0.2 % Triton; Cat# X100 Sigma-Aldrich) for 10 min at 25 °C. Cardiomyocytes were then incubated with Protein block (Dako, Victoria, Australia) to prevent unspecific antibody binding followed by Cardiac Troponin-T (cTnT 1:500; mouse polyclonal antibody; Cat# ab45932, Abcam) and Anti-Hsp60 (1:300; rabbit polyclonal antibody; Cat# ab46798, Abcam) overnight incubation at 4 °C. Cardiomyocytes were then incubated with Alexa Fluor 594 (1:200; goat anti-mouse secondary antibody; Cat# A20004, Thermo Fisher), Alexa Fluor 488 (1:200; goat anti-rabbit secondary antibody; Cat# A20000, Thermo Fisher) and DAPI (1:200; nuclear counterstain; Cat# 62248, Thermo Fisher) for 1 h at 25 °C. Coverslips containing cardiomyocytes were mounted onto glass slides using a fluorescence mounting medium (Cat# S3023, Agilent Dako) and left to dry overnight at 4 °C. Fluorescence images of mitochondria, cardiomyocytes and nucleus were captured using a Leica Thunder fluorescence microscope at 600× magnification from 10 fields of view and a total of ∼60 cardiomyocytes were analysed per biological replicate. Cells were classified as having either predominantly (>50 %) elongated (network) or fragmented mitochondria by a blinded investigator. The sum of elongated (network) or fragmented mitochondria is equal to 100 % for each condition.

### 2.9 Assessment of cell death

Cardiomyocyte death was measured using the propidium iodide (Sigma-Aldrich) cell staining method [43]. hiPSC-CM were seeded at 20,000 cells/well in clear 48-well plates containing iPSC cardiomyocyte media, and incubated at 37 °C and 5 % carbon dioxide for 24 h. hiPSC-CM were then exposed to vehicle control (0.0001 % Ultrapure Milli-Q water) or hydrogen peroxide (100 μM) ± MitoQ or dTPP (1 μM) for 1 or 48 h. Cells were then incubated with 5 μg/mL propidium iodide and 1 μg/mL Hoechst 33258 (Sigma Aldrich) in the dark at 37 °C in a humidified 5 % CO_2_ incubator for 40 min. Fluorescent images from 3 random fields of view were immediately captured using an Olympus IX71 (Tokyo, Japan) at 200× magnification. Images were later analysed using ImageJ software (Cell Counter plugin). The number of dead cells (propidium iodide) was expressed as a percentage of the total number of cells (Hoechst 33258).

### 2.10 Statistical analysis

Data represented as mean ± SEM (with individual data points shown in figures). Data analysis was carried out using one-way ANOVA with Dunnett's *post hoc* test. *P* < 0.05 was considered to be statistically significant. Statistical analysis was carried out using GraphPad Prism (version 10.0.3).

## 3 Results

### 3.1 MitoQ blunts acute oxidative stress-induced overproduction of extracellular superoxide

**Fig. 1:**
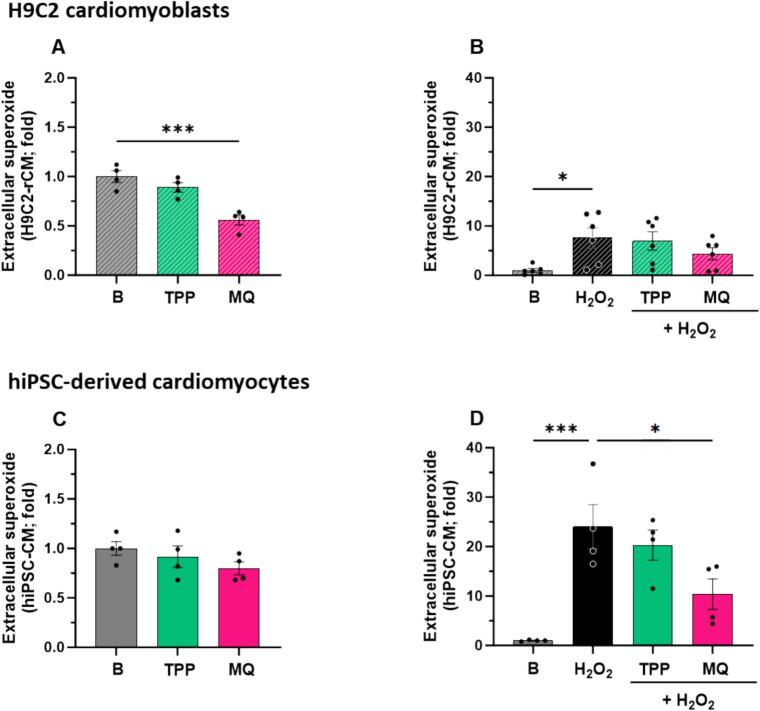
MitoQ protects against acute oxidative stress-induced overproduction of extracellular superoxide in cardiomyocytes *in vitro*. The effect of MitoQ and its dTPP control (both 1 μM) on (A) basal extracellular superoxide production (*n* = 4) and (B) acute oxidative stress (5 min H_2_O_2_ [100 μM])-stimulated extracellular superoxide production in H9C2 rat cardiomyoblasts (H9C2-rCM) (*n* = 6). The effect of MitoQ and its dTPP control (both 1 μM) on (C) basal extracellular superoxide production (n = 4) and (D) acute oxidative stress (5 min H_2_O_2_ [100 μM])-stimulated extracellular superoxide production in human induced pluripotent stem cell-derived cardiomyocytes (hiPSC-CM). (n = 4). (n) is defined as the average of 30,000 cells per biological replicate. A total of 6 independent experiments were carried out (where individual data points are biological replicates). Data represented as mean ± SEM. Data analysis was carried out using ordinary one-way ANOVA with Dunnett's *post hoc* test. *P < 0.05, ***P < 0.001 (compared to H_2_O_2_ control). B, Basal; MQ, MitoQ (Mitoquinone mesylate); dTPP, dodecyl-triphenylphosphonium; H_2_O_2_, hydrogen peroxide; H9C2-rCM, H9C2 rat cardiomyoblasts; hiPSC-CM, human induced pluripotent stem cell-derived cardiomyocytes.

The effects of MitoQ and dTPP (a molecule with the same dTPP moiety as MitoQ but without the antioxidant coenzyme Q_10_) on extracellular superoxide levels were assessed using L012 chemiluminescence in H9C2-rCM or hiPSC-CM. Under normal physiological conditions, MitoQ but not dTPP, significantly decreased extracellular superoxide levels in H9C2-rCM, compared to basal levels (*P* < 0.001, Fig. 1A). To recapitulate excess levels of superoxide observed in the failing heart [19,44], H9C2-rCM were subjected to acute exposure to hydrogen peroxide (100 μM). H9C2-rCM exposed to hydrogen peroxide induced a significant increase in extracellular superoxide production, compared to basal levels (*P* < 0.05, Fig. 1B). Elevated levels of superoxide were not significantly impacted by MitoQ or dTPP (Fig. 1B). We then sought to confirm our findings using human iPSC-derived cardiomyocytes (hiPSC-CM). Basal levels of extracellular superoxide were not significantly impacted by MitoQ or dTPP treatment (Fig. 1C). Hydrogen peroxide significantly increased extracellular superoxide production (∼25-fold) compared to basal levels (*P* < 0.001, Fig. 1D), and MitoQ, but not dTPP, blunted this effect (P < 0.05, Fig. 1D).

### 3.2 MitoQ blunts acute intracellular oxidative stress

**Fig. 2:**
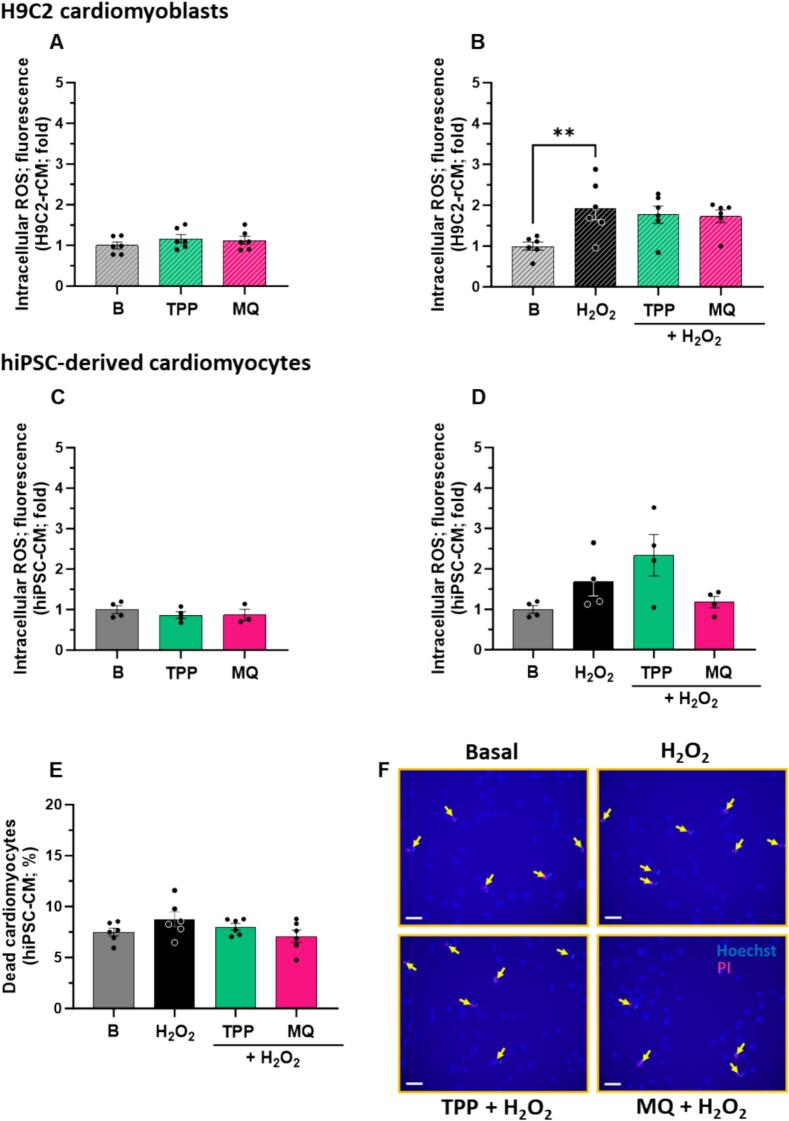
The effect of MitoQ and dTPP on acute oxidative stress-induced intracellular oxidative stress in cardiomyocytes *in vitro*. The effect of MitoQ and its dTPP control (both 1 μM) on (A) basal ROS production (*n* = 6) and (B) acute oxidative stress (5 min H_2_O_2_ [100 μM])-stimulated intracellular ROS production in H9C2 rat cardiomyoblasts (H9C2-rCM) (n = 6). The effect of MitoQ and its dTPP control (both 1 μM) on (C) basal ROS production (*n* = 4) and (D) acute oxidative stress (5 min H_2_O_2_ [100 μM])-stimulated ROS production in human induced pluripotent stem cell-derived cardiomyocytes (hiPSC-CM) (n = 4). (E) Quantification of cell death from 60 min exposure to H_2_O_2_ 100 μM ± MitoQ and dTPP (1 μM) with (F) representative propidium iodide and Hoechst staining images (scale bar = 50 μm) (n = 6). Panels A–D, (n) is defined as the average of 30,000 cells per biological replicate. Panel E, (n) is defined as the average of at least 300 cells. A total of 6 independent experiments were carried out (where individual data points are biological replicates). Data represented as mean ± SEM. Data analysis was carried out using ordinary one-way ANOVA with Dunnett's *post hoc* test. **P < 0.01 (compared to H_2_O_2_ control). B, Basal; ROS, reactive oxygen species; MQ, MitoQ (Mitoquinone mesylate); dTPP, dodecyl-triphenylphosphonium; H_2_O_2_, hydrogen peroxide; H9C2-rCM, H9C2 rat cardiomyoblasts; hiPSC-CM, human induced pluripotent stem cell-derived cardiomyocytes.

The effects of MitoQ and dTPP on intracellular oxidative stress in H9C2-rCM or hiPSC-CM were assessed using DCFDA. MitoQ and dTPP did not impact basal levels of intracellular ROS in H9C2-rCM (Fig. 2A). Hydrogen peroxide (100 μM) significantly increased intracellular ROS levels (∼2-fold) compared to basal levels in H9C2-rCM (*P* < 0.01, Fig. 2B), and this was not significantly impacted with MitoQ or dTPP treatment (Fig. 2B).

No impact on basal intracellular ROS levels was observed in hiPSC-CM with either MitoQ or dTPP treatment, compared to basal levels (Fig. 2C), indicating that physiological levels of intracellular ROS were preserved. No impact was observed with acute exposure to hydrogen peroxide alone, and in the presence of MitoQ or dTPP, compared to baseline (Fig. 2D).

Lastly, we carried out propidium iodide staining to evaluate the cytoprotective effect of MitoQ against oxidative stress-induced cell death. No differences were observed in the percentage of cell death with 1-hour exposure to hydrogen peroxide alone, and in the presence of MitoQ or dTPP, compared to baseline (Fig. 2E).

Representative images of propidium iodide staining presented in Fig. 2F. A longer exposure to oxidative stress may be required to determine whether antioxidant defence with MitoQ translates to overall cardiomyocyte protection.

### 3.3 MitoQ attenuates mitochondrial dysregulation in hiPSC-CM exposed to chronic oxidative stress

**Fig. 3:**
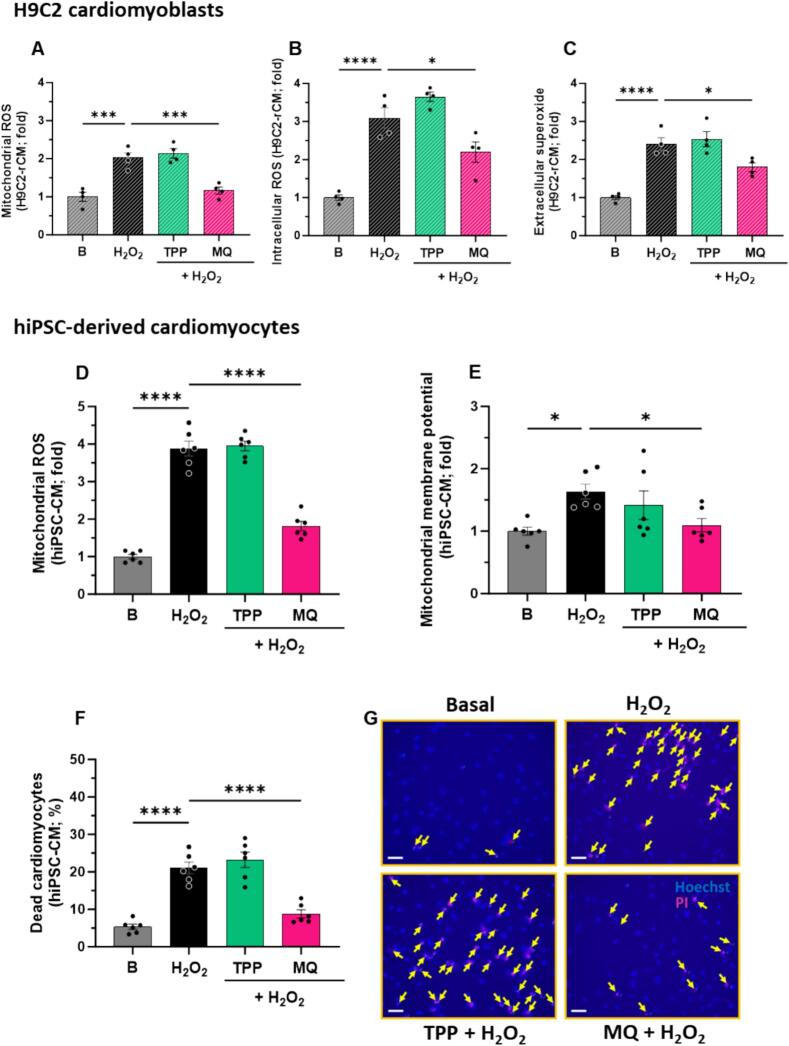
The effect of MitoQ and dTPP on chronic oxidative stress-induced mitochondrial dysregulation and cell death in cardiomyocytes *in vitro*. The effect of MitoQ and its dTPP control (both 1 μM) on (A) mitochondrial ROS (B) intracellular ROS and (C) extracellular superoxide in H9C2 rat cardiomyoblasts (H9C2-rCM) exposed to 48 h of H_2_O_2_ (100 μM). The effect of MitoQ and its dTPP control (both 1 μM) on 48 h of H_2_O_2_ (100 μM)–stimulated excess (D) mitochondrial ROS production, (E) hyperpolarised mitochondrial membrane potential and (F) cell death in human induced pluripotent stem cell-derived cardiomyocytes (hiPSC-CM). (G) representative propidium iodide and Hoechst staining images (scale bar = 50 μm). Panels A–E, (n) is defined as the average of 30,000 cells per biological replicate. Panel F, (n) is defined as the average of at least 300 cells. A total of 4 (panels A–C) or 6 (panels D–F) independent experiments were carried out (where individual data points represent biological replicates). Data analysis was carried out using ordinary one-way ANOVA with Dunnett's *post hoc* test. *P < 0.05, ***P < 0.001, ****P < 0.0001 (compared to H_2_O_2_ control). B, Basal; MQ, MitoQ (Mitoquinone mesylate); dTPP, dodecyl-triphenylphosphonium; ROS, reactive oxygen species; H_2_O_2_, hydrogen peroxide; hiPSC-CM, human induced pluripotent stem cell-derived cardiomyocytes.

We exposed hiPSC-CM and H9C2-rCM to chronic oxidative stress (48 h of exposure to 100 μM hydrogen peroxide). In H9C2-rCM, exposure to 48 h of hydrogen peroxide significantly increased mitochondrial ROS (*P* < 0.001, Fig. 3A), intracellular ROS (*P* < 0.0001, Fig. 3B) and extracellular superoxide (*P* < 0.0001, Fig. 3B) compared to basal levels. This was all blunted by MitoQ, but not dTPP, (P < 0.001, Fig. 3A; *P* < 0.05 Fig. 3B; P < 0.05 Fig. 3C).

In hiPSC-CM, an approximate 4-fold increase in mitochondrial ROS production was observed with exposure to 48 h of hydrogen peroxide, compared to basal levels (P < 0.0001, Fig. 3D). Treatment with MitoQ, but not dTPP, significantly blunted hydrogen peroxide-induced excess levels of mitochondrial ROS (P < 0.0001, Fig. 3D). The effect of MitoQ on mitochondrial membrane potential was assessed using TMRM in hiPSC-CM. Exposure to hydrogen peroxide for 48 h induced a significant increase in mitochondrial membrane potential, inducing a mitochondrial hyperpolarised state (P < 0.05, Fig. 3E). This effect was blunted by MitoQ (P < 0.05, Fig. 3E), but not dTPP. Exposure to 48 h of hydrogen peroxide significantly induced cell death by ∼15 %, compared to baseline (P < 0.0001, Fig. 3F). Interestingly, MitoQ almost completely abolished hydrogen peroxide-induced cell death (P < 0.0001, Fig. 3F). However, this effect was not observed with dTPP (Fig. 3F). Representative images of propidium iodide staining presented in Fig. 3G.

### 3.4 Both MitoQ and its mitochondrial-targeting moiety dTPP preserved mitochondrial morphology in the presence of chronic oxidative stress

**Fig. 4:**
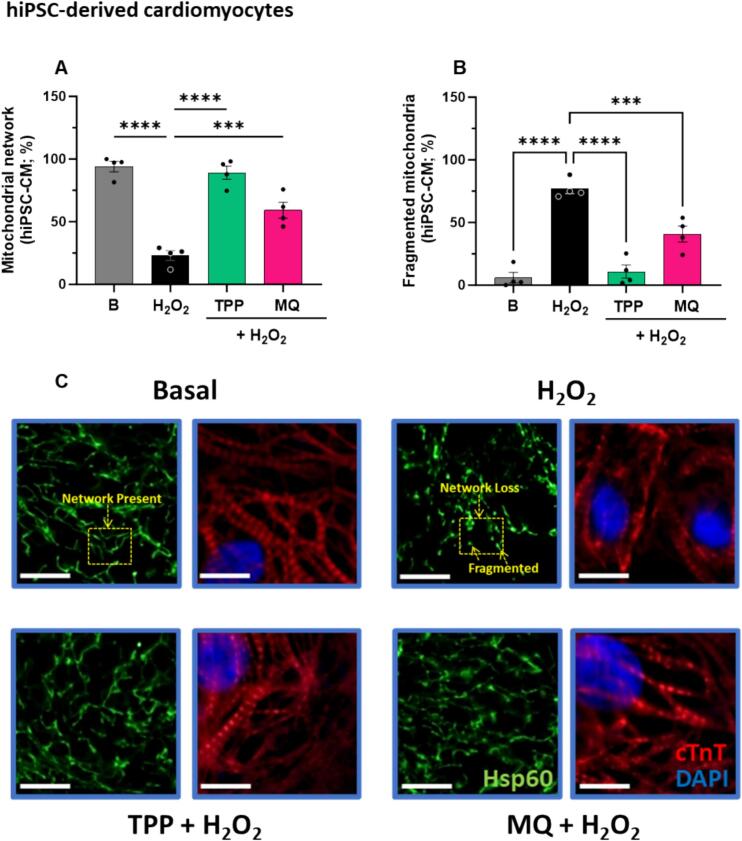
The effect of MitoQ and dTPP in chronic oxidative stress-induced adverse mitochondrial structural changes in human induced pluripotent stem cell-derived cardiomyocytes (hiPSC-CM). The effect of MitoQ and its dTPP control (both 1 μM) on mitochondrial morphological parameters including (A) network (*n* = 4) and (B) fragmentation in hiPSC-CM exposed to H_2_O_2_ (100 μM) for 48 h (n = 4). (C) Representative fluorescent images of mitochondrial morphology at 600× magnification in hiPSC-CM stained with Hsp60 (green) and cTnT (red) antibody, and DAPI (blue). Scale bar = 10 μm. Cells were classified as having either predominantly (>50 %) elongated (network) or fragmented mitochondria (loss of mitochondrial connection); the sum of elongated (network) or fragmented mitochondria is equal to 100 % for each condition. Data represented as mean ± SEM. (n) is defined as the average of at least 30 cells (where individual data points represent biological replicates). A total of 3 independent experiments were carried out. Data analysis was carried out using ordinary one-way ANOVA with Dunnett's *post hoc* test. **P < 0.01, ***P < 0.001, ****P < 0.0001 (compared to H_2_O_2_ control). B, Basal; MQ, MitoQ (Mitoquinone mesylate); dTPP, dodecyl-triphenylphosphonium; H_2_O_2_, hydrogen peroxide. (For interpretation of the references to colour in this figure legend, the reader is referred to the web version of this article.)

Hsp60 immunostaining was carried out to determine the effect of MitoQ on mitochondrial morphology. The percentage of hiPSC-CMs exhibiting a networked mitochondrial morphology was significantly decreased (to ∼25 %) in hiPSC-CMs exposed to hydrogen peroxide (100 μM) for 48 h, compared to basal (*P* < 0.0001, Fig. 4A). Interestingly, while MitoQ partially restored the mitochondrial network (to ∼60 %, *P* < 0.001, Fig. 4A), dTPP almost completely restored the mitochondrial network (to ∼90 %, P < 0.0001, Fig. 4A).

Chronic oxidative stress caused severe mitochondrial fragmentation (∼75 % increase), compared to basal (P < 0.0001, Fig. 4B). Both MitoQ and dTPP significantly prevented mitochondrial fragmentation (down to ∼40 and ∼ 9 %, respectively) in the presence of hydrogen peroxide-induced chronic oxidative stress (P < 0.0001 and *P* < 0.001, respectively, Fig. 4B).

### 3.5 MitoQ preserved mitochondrial function in the presence of chronic oxidative stress

**Fig. 5:**
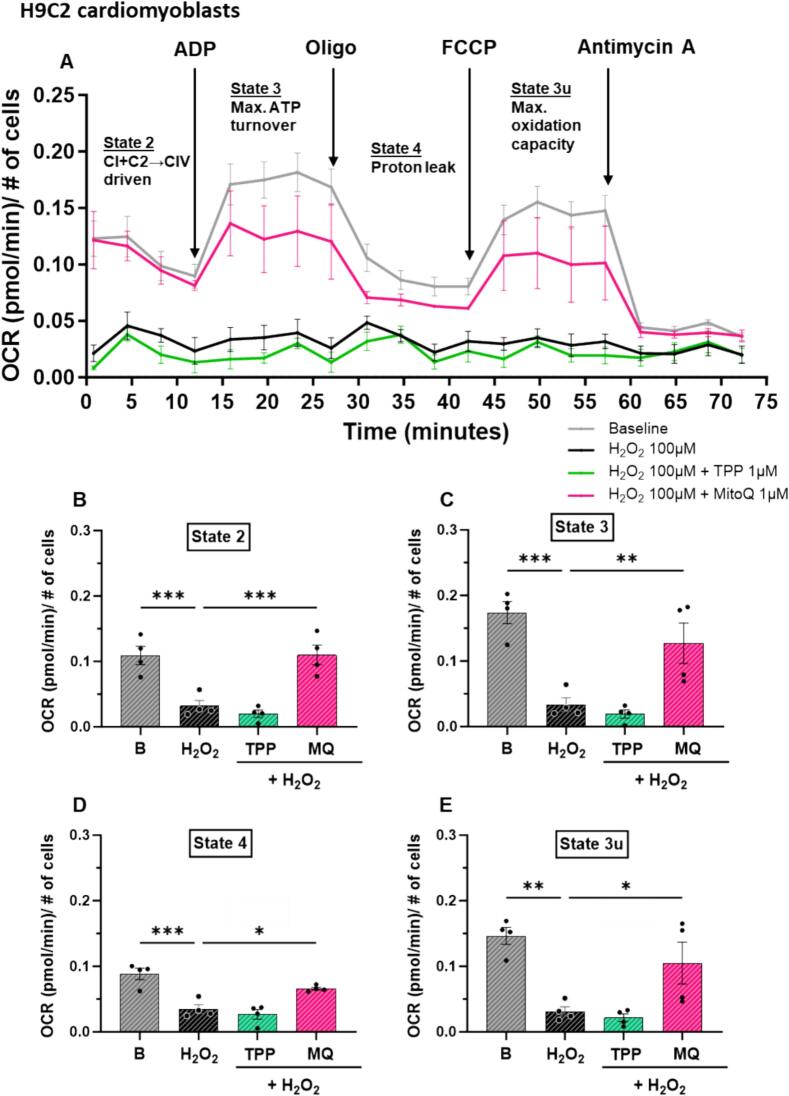
The effect of MitoQ and dTPP in chronic oxidative stress-induced adverse mitochondrial functional changes in H9C2 rat cardiomyoblasts (H9C2-rCM). (A) Representative kinetic OCR trace in H9C2-rCM. The effect of MitoQ and its dTPP control (both 1 μM) on mitochondrial functional parameters including (B) state 2 respiration (mitochondrial CI + CII driven OCR) (n = 4), (C) state 3 respiration (mitochondrial capacity to generate ATP) (D) State 4 respiration (proton leak state) (n = 4) and (E) state 3_u_ respiration (maximum mitochondrial oxidation capacity) (n = 4) in H9C2-rCM exposed to H_2_O_2_ (100 μM) for 48 h. (n) is defined as the average of at least 10,000 cells (where individual data points represent biological replicates). Data represented as mean ± SEM. Data analysis was carried out using ordinary one-way ANOVA with Dunnett's *post hoc* test. *P < 0.05, **P < 0.01, ***P < 0.001 (compared to H_2_O_2_ control). B, Basal; MQ, MitoQ (Mitoquinone mesylate); dTPP, dodecyl-triphenylphosphonium; H_2_O_2_, hydrogen peroxide; OCR, oxygen consumption rate; CI, mitochondrial complex 1; CII, mitochondrial complex 2; H9C2-rCM, H9C2 rat cardiomyoblasts.

OCR measurements were recorded to assess the impact of MitoQ on mitochondrial function in H9C2-rCM under chronic oxidative stress conditions. A representative OCR kinetic trace is presented in Fig. 5A. Several mitochondrial respiratory states (defined by Chance and [45,46]) were assessed. In a respiratory state 2 (mitochondrial complex 1 [CI] and complex 2 [CII] driven OCR), exposure to hydrogen peroxide for 48 h reduced OCR compared to basal levels (P < 0.001, Fig. 5B), but this was restored by MitoQ, but not dTPP (P < 0.001, Fig. 5B). At state 3 respiration (reflecting the capacity for a mitochondrion to generate ATP), chronic exposure to hydrogen peroxide significantly decreased mitochondria's capacity to generate ATP demonstrated by diminished OCR compared to basal (*P* < 0.001, Fig. 5C). MitoQ, but not dTPP, restored mitochondria's capacity to generate ATP (*P* < 0.01, Fig. 5C). In a proton leak state 4 respiration, OCR levels were diminished by chronic hydrogen peroxide exposure (P < 0.001, Fig. 5D), but this was normalised back almost to basal levels by MitoQ treatment (*P* < 0.05, Fig. 5D). In a state 3_u_ respiration (assessing the maximum mitochondrial oxidation capacity), OCR levels were significantly lower in the hydrogen peroxide group, compared to basal levels (P < 0.01, Fig. 5E), and MitoQ partially restored these levels (P < 0.05, Fig. 5E).

## 4 Discussion

Heart failure is a lethal form of cardiometabolic disease accounting for the highest cause of death worldwide [47]. The current challenge in managing heart failure is that conventional treatments focus on symptom relief rather than specifically targeting the underlying pathological mechanisms [48,49]. Superoxide is a reactive molecule that can generate several other forms of ROS that drive the remodelling observed in the failing heart [49,50]. It is well known that increased ROS production can cause mitochondrial dysregulation and cell death, which contribute to cardiac remodelling in heart failure [51,52]. Thus, targeting excess ROS-induced oxidative stress is an attractive strategy for treating heart failure [44,53].

We sought to validate the protective effects of the mitochondria-targeted antioxidant MitoQ in cardiomyocytes (both H9C2-rCM and hiPSC-CM) exposed to oxidative stress. In the present study, we demonstrate that MitoQ treatment mitigates excess levels of extracellular superoxide, cellular and mitochondrial ROS, and preserves mitochondrial network and membrane potential. Consistent with our study, elevated ROS was previously observed in oxidative stress-induced (*via* isoproterenol exposure) neonatal rat cardiomyocytes, which was accompanied by decreased sirtuin 3/mitochondrial ROS dismutase (SERT3/SOD2) interaction leading to SOD2 inactivation and increased cell size; all of which were attenuated with MitoQ treatment [54]. Interestingly, MitoQ blunted excess mitochondrial ROS in our study, suggesting that MitoQ may be indirectly removing excess mitochondrial superoxide by preventing SOD2 inactivation and thus maintaining redox regulation. MitoQ also previously exhibited cytoprotection by blunting ROS levels, normalising mitochondrial membrane potential and reducing the level of cellular apoptosis, and this protection was associated with increased expression of PINK1/Parkin, in high glucose and fatty acid-treated H9C2-rCM [55]. Given that the interaction between PINK 1/Parkin governs mitochondrial quality control, MitoQ may also be acting through this pathway to restore mitochondrial regulation and redox state [55,56]. These findings together with our study suggest that MitoQ may be acting on multiple targets (including mitochondrial and redox regulatory pathways) to attenuate cardiac remodelling in oxidative stress conditions. Thus, targeting oxidative stress with MitoQ may be an attractive strategy to prevent cardiomyocyte injury in the failing heart.

Mitochondrial structural and functional dysregulation play important roles in heart failure as mitochondria are major sites of superoxide production in the myocardium [10,50]. In the present study, we show that hiPSC-CMs exposed to oxidative stress exhibited increased mitochondrial membrane potential leading to hyperpolarisation, and this was associated with excess mitochondrial ROS production. It has been proposed that elevated mitochondrial membrane potential in the myocardium promotes higher electron flow, surpassing the capacity of electron carriers in the mitochondrial respiratory chain, which can result in singlet electron leakage and subsequent production of superoxide [51,57,58]. Indeed, electrons prematurely exiting the respiratory chain can reduce mitochondrial functional capacity [59], as demonstrated in H9C2-rCM exhibiting lower OCR in respiration states 2, 3, 3u, and 4 from chronic exposure to oxidative stress. This can lead to lower overall bioenergetic production exhibited in heart failure [60]. In addition, increased cardiac mitochondrial ROS levels are also associated with injury to the inner mitochondrial membrane cristae [61]. We now show that MitoQ exhibits cytoprotection against both oxidative stress-induced excess mitochondrial ROS and hyperpolarisation. A previous study demonstrated that normalising mitochondrial ROS levels with MitoQ supplementation was accompanied by restoration of Mitofusin-2 regulation and improvements in mitochondrial structural integrity in the myocardium of mice subjected to pressure overload-induced cardiac dysfunction [30]. Given that endogenous coenzyme Q_10_ functions to shuttle electrons between the mitochondrial electron transport chain complexes, MitoQ may also be replenishing these electron carriers to prevent electron leakage in the mitochondrial respiratory chain and subsequent mitochondrial ROS production [62]. Therefore, restoring the coenzyme Q_10_ pool may contribute to improving the mitochondrial respiratory chain function and normalising the mitochondrial membrane potential [63].

In the present study, both MitoQ and dTPP exhibited mitochondrial structural protection by preserving the mitochondrial network and preventing mitochondrial fragmentation. Interestingly, this pro-fusion effect is larger with the dTPP treatment alone compared to MitoQ, suggesting that the coenzyme Q_10_ moiety is partially suppressing the protection of the dTPP moiety. Another possibility is mitochondrial accumulation differences between MitoQ and dTPP. Both MitoQ (dTPP+CoenzymeQ_10_) and dTPP are pulled into the mitochondria *via* charge differences derived from the mitochondrial membrane potential [22,64]. Given that both compounds have the same strong positively charged cation (phosphonium), the size difference between MitoQ (678.8 g/mol) and dTPP (483.5 g/mol) may have resulted in a greater accumulation of dTPP in the mitochondria [65]. A previous study demonstrated equivalent protection with MitoQ or dTPP supplementation in whole-body metabolism in obese mice, also suggesting that the dTPP cation was responsible for these effects [24]. The lipophilic cation dTPP (10‑carbon chain linked to 3 phenolic aromatic rings and a positively charged phosphate ion) was originally engineered to facilitate the uptake of the antioxidant coenzyme Q_10_ into the mitochondria to exert protection [22], however, our data demonstrate that the dTPP moiety may have a secondary protective effect in addition to its intended primary role as a carrier for coenzyme Q_10_. Phenolic-containing antioxidants are known to inhibit lipid peroxidation [66,67], therefore it is possible that the dTPP may also act to target reactive lipid species to preserve the mitochondrial phospholipid membrane.

Cardiac apoptosis is a key pathway that leads to contractile dysfunction in heart failure [68]. Here, we demonstrate that oxidative stress-induced cell death in hiPSC-hCMs was almost completely abolished by MitoQ treatment. A similar protective role of MitoQ has been observed in H9C2-rCM subjected to ischaemia reperfusion injury, doxorubicin-induced cardiac remodelling, angiotensin II-induced apoptosis and triptolide-induced cardiotoxicity [55,[69], [70], [71], [72]]. It has been proposed that dysfunctional mitochondria in the myocardium exhibit elevated ROS generation, fragmentation and membrane potential dysregulation, which promotes apoptogenic pathways by releasing caspase proteins, resulting in cell death [29,51,73,74]. Given these studies, we speculate that the protection exhibited by MitoQ against cell death in this study may be in part due to the inhibition of the mitochondrial-mediated apoptotic pathways by preventing excess ROS, mitochondrial fragmentation and hyperpolarisation [[75], [76], [77]].

For the first time, we have revealed the cytoprotective effects of MitoQ treatment in oxidative stress-induced human cardiac myocytes by normalising excess ROS levels, mitochondrial hyperpolarisation and attenuating cell death. This effect is likely due to the coenzyme Q_10_ component of MitoQ. We also demonstrate that the dTPP moiety of MitoQ preserved mitochondrial structural integrity. These results suggest that both components of MitoQ (coenzyme Q_10_ and dTPP) provide complementary cytoprotection in several mitochondrial parameters. Findings from this study suggest that therapeutically targeting the mitochondria with MitoQ in the setting of oxidative stress may be an attractive strategy to mitigate adverse cardiac remodelling.

## 5 Limitations

We acknowledge that our study had several limitations, which are discussed here. We specifically sought to explore the independent role of hydrogen peroxide in the pathological derangements of heart failure given that patients exhibit increased levels of hydrogen peroxide [13,78,79]. Given that MitoQ is a ROS scavenger, we wanted to confirm that the effect of MitoQ exhibited in this study was preventing the hydrogen peroxide-induced cardiac remodelling in heart failure. We acknowledge that other methods generate more pathologically relevant stimuli in the induction of oxidative stress in heart failure such as exposure to Angiotensin II, isoproterenol and/or hypoxia/reoxygenation, which will be explored in a follow-up study [80]. The focus of this study was the impact of MitoQ on oxidative stress-induced cardiomyocyte mitochondrial remodelling. However, we do acknowledge that additional experiments will help clarify the effect of dTPP. The mechanistic insight of dTPP will be further explored in a future study. Additionally, we recognise that the H9C2 cell line in its undifferentiated state lacks structural and functional similarities exhibited in adult cardiomyocytes.

## 6 Conclusions

In conclusion, for the first time, we have revealed the cytoprotective effects of MitoQ treatment in oxidative stress-induced human cardiac myocytes by normalising excess ROS levels, mitochondrial hyperpolarisation and attenuating cell death. This effect is likely due to the coenzyme Q_10_ component of MitoQ. We also demonstrate that the dTPP moiety of MitoQ preserved mitochondrial structural integrity. These results suggest that both components of MitoQ (coenzyme Q_10_ and dTPP) provide complementary cytoprotection in several mitochondrial parameters. Findings from this study suggest that therapeutically targeting the mitochondria with MitoQ in the setting of oxidative stress may be an attractive strategy to mitigate adverse cardiac remodelling.

The following are the supplementary data related to this article.

Supplementary Table 1 Source information for cell culture media, supplements and experimental reagents. Supplementary Table 1

Supplementary Fig. 1 **A)** cTnT (specific marker of cardiomyocytes) staining of differentiated iPSCs. **B)** Representative image of cTnT+ cells (green) and DAPI (blue) (scale bar = 50 μm). Differentiated iPSCs used for experiments exhibited cTnT+ of 95 % or above. hiPSC-hCM, human induced pluripotent stem cell-derived cardiomyocytes; cTnT, Cardiac troponin T. Supplementary Fig. 1

## CRediT authorship contribution statement

**Alex M. Parker:** Writing – review & editing, Writing – original draft, Methodology. **Jarmon G. Lees:** Writing – review & editing, Supervision, Project administration, Investigation. **Mitchel Tate:** Writing – review & editing, Supervision, Methodology, Investigation. **Ren J. Phang:** Writing – review & editing, Methodology, Investigation. **Anida Velagic:** Writing – review & editing, Methodology, Investigation. **Minh Deo:** Writing – review & editing, Methodology, Investigation. **Tayla Bishop:** Methodology, Investigation, Formal analysis. **Thomas Krieg:** Writing – review & editing, Resources, Methodology, Investigation. **Michael P. Murphy:** Writing – review & editing, Resources, Methodology, Investigation. **Shiang Y. Lim:** Writing – review & editing, Supervision, Resources, Project administration, Methodology, Investigation, Funding acquisition. **Miles J. De Blasio:** Writing – review & editing, Supervision, Project administration, Methodology, Investigation, Funding acquisition. **Rebecca H. Ritchie:** Writing – review & editing, Validation, Supervision, Project administration, Methodology, Investigation, Funding acquisition.

## Funding

This work was supported by the 10.13039/501100000925National Health and Medical Research Council of Australia (NHMRC), including an NHMRC project grant APP1158013 (to RHR, MPM and MJD) and NHMRC Fellowship support (to RHR, Senior Research Fellowship APP1059960), as well as the 10.13039/501100000971Diabetes Australia Research Program (DARP, Y24G-DEBM), Stafford Fox Medical Research Foundation, St Vincent's Institute of Medical Research and Operational Infrastructure Support from the Victorian State Government's Department of Innovation, Industry and Regional Development to the Baker Heart & Diabetes Institute.

## Declaration of competing interest

The authors declare the following financial interests/personal relationships which may be considered as potential competing interests: Rebecca H. Ritchie reports equipment, drugs, or supplies were provided by Antipodean Pharmaceuticals (Auckland, New Zealand). If there are other authors, they declare that they have no known competing financial interests or personal relationships that could have appeared to influence the work reported in this paper.
