# Supplementary material for: MitoQ Protects Against Oxidative Stress-Induced Mitochondrial Dysregulation in Human Cardiomyocytes
Source: J Mol Cell Cardiol Plus. 2025 Jun 26;13:100469. doi: 10.1016/j.jmccpl.2025.100469 (PMC12271626; doi:10.1016/j.jmccpl.2025.100469)
Supplement: Supplementary Table 1 — Source information for cell culture media, supplements and experimental reagents. [file mmc1.docx]

| **Cell culture media** | **Catalogue number** | **Source** |
| --- | --- | --- |
| DMEM | 11965092 | Thermo Fisher Scientific |
| TeSR-E8 | 5990 | Stem Cell Technologies |
| RPMI 1640 | 11875093 | Thermo Fisher Scientific |
| DMEM-F12 GlutaMax | 10565042 | Sigma-Aldrich |
| HBSS | 14025092 | Thermo Fisher Scientific |
| PBS | P4417 | Sigma Aldrich |
| Krebs buffer | K4002 | Sigma Aldrich |
|  |  |  |
| **Cell culture supplements** | **Catalogue number** | **Source** |
| FBS | 10099141 | Thermo Fisher Scientific |
| Penicillin/streptomycin | 15140122 | Thermo Fisher Scientific |
| Glucose | A2494001 | Thermo Fisher Scientific |
| B27 (insulin free) | A1895601 | Thermo Fisher Scientific |
| CHIR99021 | 13122 | Cayman Chemical |
| Matrigel (growth factor reduced) | CLS356231 | Sigma Aldrich |
| Y-27632 dihydrochloride | 1254 | Tocris Bioscience |
| IWP2 | 3533 | Tocris Bioscience |
| Ascorbic acid | A92902 | Sigma Aldrich |
| Fetal calf serum | 12006C | Sigma Aldrich |
| MEM non-essential amino acids | M7145 | Sigma Aldrich |
| 2-mercaptoethanol | 21985023 | Thermo Fisher Scientific |
| lactate | 1614308 | Sigma Aldrich |
| B27 | A3582801 | Thermo Fisher Scientific |
|  |  |  |
| **Experimental reagents** | **Catalogue number** | **Source** |
| L-012 | SML2236 | Sigma Aldrich |
| DCFDA | D399 | Thermo Fisher Scientific |
| MitoSOX red | M36008 | Thermo Fisher Scientific |
| TMRM | T668 | Thermo Fisher Scientific |
| Hsp60 | ab46798 | Abcam |
| cTnT | ab45932 | Abcam |
| Anti-Hsp60 | ab46798 | Abcam |
| Alexa Fluor 594 | A20004 | Thermo Fisher Scientific |
| Alexa Fluor 488 | A20000 | Thermo Fisher Scientific |
| DAPI | 62248 | Thermo Fisher Scientific |
| Propidium iodide | P4170 | Sigma Aldrich |
| Hoechst 33258 | 94403 | Sigma Aldrich |
| Hydrogen peroxide | 605852 | Gold Cross |
| dTPP | 172626 | Sigma Aldrich |
| Triton X-100 | X100 | Sigma-Aldrich |

**Supplementary Table 1.** Source information for cell culture media, supplements and experimental reagents.
