# Supplementary material for: MitoQ Protects Against Oxidative Stress-Induced Mitochondrial Dysregulation in Human Cardiomyocytes
Source: J Mol Cell Cardiol Plus. 2025 Jun 26;13:100469. doi: 10.1016/j.jmccpl.2025.100469 (PMC12271626; doi:10.1016/j.jmccpl.2025.100469)
Supplement: Supplementary Fig. 1 — A) cTnT (specific marker of cardiomyocytes) staining of differentiated iPSCs. B) Representative image of cTnT+ cells (green) and DAPI (blue) (scale bar = 50 μm). Differentiated iPSCs used for experiments exhibited cTnT+ of 95 % or above. hiPSC-hCM, human induced pluripotent stem cell-derived cardiomyocytes; cTnT, Cardiac troponin T. [file mmc2.docx]

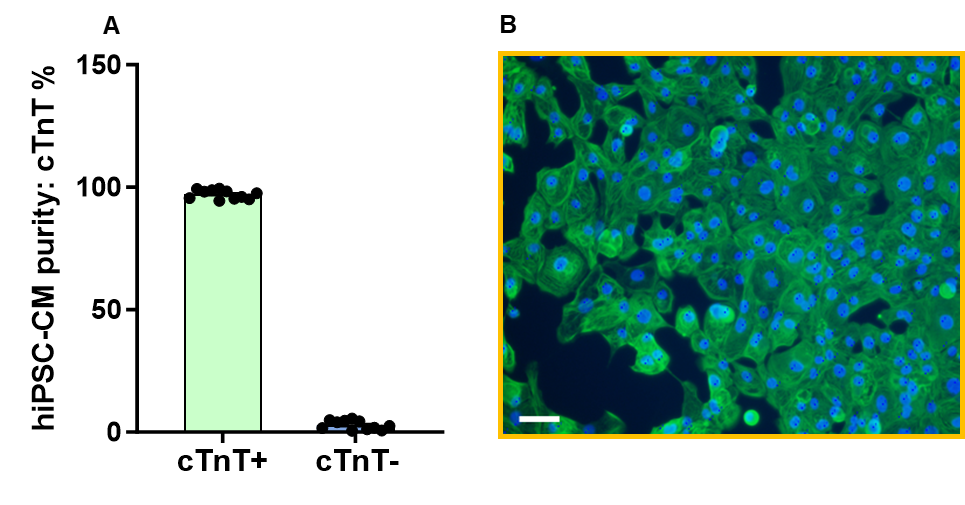


**Supplementary Figure 1. A)** cTnT (specific marker of cardiomyocytes) staining of differentiated iPSCs. **B)** Representative image of cTnT+ cells (green) and DAPI (blue) (scale bar = 50 μm). Differentiated iPSCs used for experiments exhibited cTnT+ of 95% or above. hiPSC-hCM, human induced pluripotent stem cell-derived cardiomyocytes; cTnT, Cardiac troponin T.
